# Supplementary material for: Rod-Shaped Starch from Galanga: Physicochemical Properties, Fine Structure and In Vitro Digestibility
Source: Foods. 2024 Jun 6;13(11):1784. doi: 10.3390/foods13111784 (PMC11171565; doi:10.3390/foods13111784)
Supplement: Supplementary file 1 [file foods-13-01784-s001.zip › foods-2993381-supplementary.pdf]

Table S1. Granule size distribution parameters of three rhizome starches [A].

Samples	$d_{(0.1)}$ [B]	$d_{(0.5)}$	$d_{(0.9)}$	D[4, 3]	D[3, 2]
AOS	8.81 ± 0.10[a]	16.38 ± 0.11[a]	26.51 ± 0.10[b]	16.84 ± 0.23[a]	9.61 ± 0.12[a]
AGS	6.54 ± 0.12[b]	15.88 ± 0.18[b]	27.87 ± 0.12[a]	16.57 ± 0.17[a]	9.21 ± 0.11[b]
ZOS	2.48 ± 0.10[c]	9.69 ± 0.10[c]	15.80 ± 0.11[c]	9.69 ± 0.11[b]	5.68 ± 0.20[c]

A $d_{(0.1)}$, 10% of the total particles exhibited a size less than this value (μm); $d_{(0.5)}$, 50% of the total particles exhibited a size less than this value (μm); $d_{(0.9)}$, 90% of the total particles exhibited a size less than this value (μm); D[4, 3], volume weight mean; D[3, 2], surface weight mean. B Each datum reported is the means ± S.D. (n = 3). In the same column with different letters indicated differ significantly ($p < 0.05$).
